# Milk Production and Energetic Metabolism of Heat-Stressed Dairy Goats Supplemented with Propylene Glycol

**DOI:** 10.3390/ani10122449

**Published:** 2020-12-21

**Authors:** Soufiane Hamzaoui, Gerardo Caja, Xavier Such, Elena Albanell, Ahmed A. K. Salama

**Affiliations:** Group of Research in Ruminants (G2R), Animal and Food Sciences Department, Universitat Autònoma de Barcelona, Bellaterra, 08193 Barcelona, Spain; sofianina2000@yahoo.fr (S.H.); gerardo.caja@uab.cat (G.C.); xavier.such@uab.cat (X.S.); elena.albanell@uab.cat (E.A.)

**Keywords:** heat load, glucogenic precursors, lactation, blood metabolites, dairy goats

## Abstract

**Simple Summary:**

Heat stress (HS) causes significant milk production losses and negatively impacts animal health. Previous research indicated that heat-stressed goats eat less and degrade their skeletal muscles to cover shortages in nutrient requirements. Propylene glycol (PG) is a glucogenic precursor and may be a remedy for energy shortage in HS situations. In the current study, dairy goats were exposed to thermoneutral (TN; 15 to 20 °C) or HS (12 h/d at 37 °C and 12 h/d at 30 °C) conditions. In each ambient temperature, goats were fed a control diet (CO) or the same diet supplemented with PG. Goats supplemented with PG gained more body weight, and experienced greater blood glucose and insulin levels compared to CO goats. However, supplementation with PG depressed feed intake and milk fat content. In conclusion, feeding propylene glycol was useful to reduce body weight losses typically observed under HS conditions, but did not improve milk yield or milk composition.

**Abstract:**

Heat-stressed dairy animals increase their reliance on glucose. This elevated glucose demand is partially met by increasing the conversion of glucogenic amino acids (AA) in the liver. Propylene glycol (PG) is a glucogenic precursor and was not tested in dairy goats under thermoneutral (TN) and heat stress (HS) conditions simultaneously. We hypothesize that if HS-goats are fed with PG, they would get more glucose and consequently spare more glucogenic AA for milk protein synthesis rather than gluconeogenesis. Eight multiparous dairy goats (40.8 ± 1.1 kg body weight; 84 ± 1 days in milk) were used in a replicated 4 × 4 Latin square design of 4 periods; 21 d each (14 d adaptation, 5 d for measurements, and 2 d of transition). Goats were allocated to one of 4 treatments in a 2 × 2 factorial arrangement. Factors were control (CO) without PG or 5% of PG, and thermoneutral (TN; 15 to 20 °C) or heat stress (HS; 12 h/d at 37 °C and 12 h/d at 30 °C) conditions. Feed intake, rectal temperature, respiratory rate, milk yield, milk composition, and blood metabolites were measured. Compared to TN, HS goats had lower (*p* < 0.01) feed intake (–34%), fat-corrected milk (–15%), and milk fat (–15%). Heat-stressed goats also tended (*p* < 0.10) to produce milk with lower protein (–11%) and lactose (–4%) contents. Propylene glycol increased blood glucose (+7%; *p* < 0.05), blood insulin (+37%; *p* < 0.10), and body weight gain (+68%; *p* < 0.05), but decreased feed intake (–9%; *p* < 0.10) and milk fat content (–23%; *p* < 0.01). Furthermore, blood non-esterified fatty acids (–49%) and β-hydroxybutyrate (–32%) decreased (*p* < 0.05) by PG. In conclusion, supplementation of heat-stressed dairy goats with propylene glycol caused milk fat depression syndrome, but reduced body weight loss that is typically observed under HS conditions. Supplementation with lower doses of PG would avoid the reduced feed intake and milk fat depression, but this should be tested.

## 1. Introduction

Compared to thermoneutral (TN) conditions, dairy goats under heat stress (HS) conditions experience lower dry matter (DM) intake and milk yield with a significant depression in milk fat and protein contents [1,2]. This decrease in milk fat and protein contents under HS conditions was widely observed also in dairy cows [3]. The reduction in milk yield and its components under HS conditions is accompanied by a down-regulation in milk fat and protein genes, and up-regulation of apoptosis genes in the mammary gland [4,5].

Under HS conditions, DM intake decreases by 25 to 40% in dairy goats, causing a negative energy balance and body weight (BW) loss [1,6]. Paradoxically, this significant reduction in DM intake is not accompanied by body fat mobilization, as blood non-esterified fatty acids (NEFA) levels did not vary between HS and TN cows [3] or goats [1,6]. Heat-stressed dairy goats are also able to keep similar blood glucose [2,6] despite the reduced feed intake and the apparent blockage of using fatty acids as compensatory energy source. Alternatively, it seems that HS-goats catabolize their muscles (protein mobilization) and use glucogenic AA to keep blood glucose levels [2,4].

Blood glucose is the only precursor for lactose synthesis, and lactose is the primary osmotic regulator of milk synthesis [7]. During established lactation, milk synthesis is not limited by glucose availability [8]. However, glucose availability has been thought to limit milk yield during HS [3]. This is because heat-stressed animals suffer some degree of inflammation as they have a compromised intestinal barrier function [3,9], which results in intestinal-derived toxins infiltration that induces immune response. Consequently, glucose availability is significantly important because the activation of the immune system consumes large amounts of glucose [10]. Given the importance of glucose for milk synthesis and the immune system, it is logical to hypothesize that if HS-goats are fed with glucogenic component, they would get more glucose and reduce muscle catabolism, which would spare more AA for milk protein synthesis rather than gluconeogenesis.

Propylene glycol (PG) is a glucogenic precursor that is either rapidly absorbed from the rumen and converted to glucose in the liver, or partially metabolized to propionate in the rumen before being absorbed and converted to glucose by gluconeogenesis [11]. Several studies in dairy cows have shown that an oral drench of PG is effective in increasing blood glucose and decreasing blood NEFA and β-hydroxybutyrate (BHB) [11,12]. The few available studies in dairy goats [13,14] showed that PG supplementation during the peripartal period resulted in increased blood glucose levels with no effects on milk yield or milk composition.

As pointed out by Joy et al. [15], an increment in cortisol levels in response to HS causes protein mobilization and catabolism to provide glucogenic AA for gluconeogenesis. Therefore, our hypothesis was that feeding propylene glycol would increase blood glucose and reduce the dependance on AA as energy source. This would result in reducing muscle catabolism and/or sparing AA for milk protein synthesis. The aim of the current study was to evaluate the lactational and metabolic responses of dairy goats to PG supplementation under TN and HS conditions. As far as we know, PG was not tested in dairy goats under TN and HS conditions simultaneously.

## 2. Materials and Methods

Animal care conditions and management practices agreed with the procedures stated by the Ethical Committee of Animal and Human Experimentation of the Universitat Autònoma de Barcelona (Bellaterra, Spain; CEEAH reference 771) and the codes of recommendations for the welfare of livestock of the Ministry of Agriculture, Food and Environment of Spain.

### 2.1. Animals, Treatments, and Management Conditions

Eight multiparous Murciano-Granadina dairy goats (84 ± 1 days of lactation, 2.00 ± 0.04 L/d of milk yield; 40.8 ± 1.1 kg BW) with healthy and symmetrical udders were used from the herd of the experimental farm of the Universitat Autònoma de Barcelona. Goats were used in a replicated 4 × 4 Latin square design with 4 periods; 21 d each (14 days adaptation, 5 days for measurements and 2 days transition between periods). When goats were exposed to HS conditions, the temperature was increased gradually throughout the first 2 days (1 day at 25 °C, 1 day at 30 °C). Goats were allocated to one of 4 treatments in a 2 × 2 factorial arrangement. Factors were control (CO) without propylene glycol or 5% of propylene glycol (PG), and thermoneutral (TN; 15 to 20 °C and 40 to 65% relative humidity throughout the day) or heat stress (HS; from 09:00 to 21:00 h at 37 °C and from 21:00 to 09:00 h at 30 °C with 45 ± 5% relative humidity) conditions. This resulted in 4 treatment combinations: TN-CO, TN-PG, HS-CO, and HS-PG. The PG goats received the propylene glycol from day 1 to 19 of each period (14 days adaptation and 5 days for measurements). Both TN and HS goats were in 2 adjacent rooms with identical facilities and management conditions. Throughout the experiment (December to March), the temperature for TN goats was maintained with the help of electric heaters equipped with a thermostat (3.5 kW; General Electric, Barcelona, Spain). The room of HS goats was provided with a temperature and humidity controlling system (CAREL Controls Ibérica, Barcelona, Spain). A continuous 90 m^3^/h air turnover was maintained throughout the experiment.

Data of environmental temperature and humidity for TN and HS goats were recorded every 10 min throughout the experiment by a data logger (Opus 10, Lufft, Fellbach, Germany). The temperature humidity index (THI) values were calculated according to NRC [16]: THI = (1.8 × T_db_ + 32) − [(0.55 − 0.0055 × RH) × (1.8 × T_db_ − 26.8)], where T_db_ is the dry bulb temperature (°C) and RH is the relative humidity (%). The THI values for TN varied between 59 and 66 throughout the day, whereas for HS, the THI values were 86 and 78 for the day and night, respectively. Silanikove and Koluman [17] proposed risk classes of HS for dairy goats according to the value of THI. According to their classification, our HS goats were supposed to be in the “danger” (85 ≤ THI < 90) and “normal” (THI < 80) stress levels during the day and night, respectively.

The total mixed ration was formulated according to INRA [18] based on the expected performance of the TN-CO goats (2.0 DM intake and 2.0 kg/d milk yield). The expected daily requirements were 1.51 UFL and 160 g PDI. According to the nutritive value of the ration (Table 1), 2 kg DM intake would provide goats with 1.64 UFL and 200 g PDI. Consequently, there were safety margins of 6 and 25% for energy and protein requirements, respectively. The ration consisted of alfalfa hay 60.4%, ground barley grain 15%, beet pulp 9.1%, ground corn grain 7.5%, soybean meal 3%, sunflower meal 3%, molasses 1%, salt 0.6%, sodium bicarbonate 0.2%, and CVM for goats 0.2%. Chemical composition and nutritive value are shown in Table 1. The nutritive value was calculated according to the INRA [18]. Mineral and vitamin blocks were freely available for each goat. The PG (1,2-propanediol—USP, LABIANA Life Sciences S.A.U. Terrassa, Barcelona, Spain) was thoroughly mixed daily with the ration. Equivalent amount of water was mixed with the CO rations.

Goats were milked once daily (08:00 h) with a portable milking machine (Westfalia-separator Ibérica, Granollers, Spain). Milking was conducted at a vacuum pressure of 42 kPa, a pulsation rate of 90 pulses/min, and a pulsation ratio of 66%. The milking routine included cluster attachment without udder preparation or teat cleaning, machine milking, machine stripping before cluster removal, and teat dipping in an iodine solution (P3-ioshield, Ecolab Hispano-Portuguesa, Barcelona, Spain).

### 2.2. Sample Collection, Analyses, and Measurements

Rectal temperatures and respiration rates were recorded at 08:00, 12:00, and 17:00 h. The rectal temperature was measured by a digital clinical thermometer (Model ICO Technology “mini color” Barcelona, Spain; 32 to 43.9 ± 0.1 °C). The respiration rate was measured by counting the inhalations and exhalations for 60 s with the aid of a digital chronometer (Model 900400; Deltalab, Barcelona, Spain).

Goats were weighted in 2 consecutive days at the start and end of each experimental period using electronic scale (Tru-Test AG500 Digital Indicator, Auckland, New Zealand; accuracy ± 20 g) to measure the change in BW. Additionally, BW values were used to calculate net energy balance using the following equation: energy balance = net energy intake – (NE_M_ + NE_L_).

Net energy for maintenance (NE_M_) was calculated using the following equation: NE_M_ = (0.0406 × BW^0.75^) according to INRA [18]. Maintenance costs were increased by 30% for HS goats as recommended by NRC [19]. Net energy for lactation (NE_L_) was calculated by using the following equation: NE_L_ = milk yield × [0.389 + 0.0052 (fat, g/kg – 35) + 0.0029 × (protein, g/kg – 31)] according to INRA [18]. For PG goats, NE_L_ value of 4.7 Mcal/kg was applied for PG [20].

Feed intake and water consumption (accuracy: ± 20 g) were recorded daily throughout the experiment. Feed samples were collected daily during measurement days of each period and were ground through a 1-mm stainless steel screen, and then analyzed for DM, ADF, NDF, and ash content according to analytical standard methods [21]. The Dumas method (Leco analyzer, LECO Corp., St. Joseph, MI, USA) was used for N determinations and CP was calculated as percentage of N × 6.25.

Milk yield of individual goats was recorded daily throughout the 5-days measurement period. A milk sample of approximately 50 mL was collected for 2 consecutive days during the measurement days of each period and were preserved with an antimicrobial tablet (Bronopol, Broad Spectrum Microtabs II, D&F Control Systems Inc., San Ramon, CA, USA) at 4 °C until analysis. Milk samples were analyzed for major components (total solids, fat, protein, and lactose) using medium infrared spectrophotometry (MilkoScan FT2, Foss, Hillerød, Denmark), and somatic cell count using an automatic cell counter (Fossomatic 5000, Foss, Hillerød, Denmark) previously calibrated for goat milk. Yield of protein, fat, and lactose were calculated using the corresponding milk yields for each sampling.

Blood samples were taken at day 5 of each measurement period from the jugular vein into vacutainers (Venoject, Leuven, Belgium) before milking and the morning feeding. Plasma was obtained by centrifugation of whole blood for 15 min at 1500× *g*, and stored at –20 °C for the analysis of NEFA, BHB, insulin, and lactate. The NEFA were analyzed by the colorimetric enzymatic test ACS-ACOD method using a commercial kit (Wako Chemicals, Neuss, Germany). The BHB was determined by kinetic enzymatic method using commercial kit (RANBUT, Randox^®^, Crumlin, UK). Insulin was measured by a ELISA type sandwich using a commercial kit (Mercodia Ovine Insulin ELISA, Mercodia^®^; Uppsala, Sweden) and the lactate was determined by enzymatic method (Olympus System Reagent^®^, Beckman Coulter^®^, Nyon, Switzerland). In addition, whole blood without anticoagulants was collected and a single drop was immediately applied to disposable cartridges (iSTAT CG8^+^ and Crea cartridges, Abbott Point of Care Inc., Princeton, NJ, USA). Then, the cartridge was inserted into an i-STAT handheld analyzer maintained at room temperature (22 °C), and the results of pH, electrolytes, hematocrit, hemoglobin, glucose, urea, and creatinine were obtained.

### 2.3. Statistical Analyses

Data were analyzed by the PROC MIXED for repeated measurements of SAS v. 9.1.3 (SAS Institute Inc., Cary, NC, USA). The statistical mixed model contained the fixed effects of the temperature (TN and HS), dietary supplementation (CO and PG), measurement day (day 1 to 5), period (1 to 4); the random effect of the animal; the interactions of temperature × supplementation, temperature × period, supplementation × period; and the residual error. For the data of rectal temperature and respiratory rate measured at 09:00, 12:00, and 17:00 h, a fixed factor of the hour of day was added to the model. For the data of blood metabolites and changes of BW, the PROC MIXED was used without repeated measures, and consequently the measurement day effect was removed from the model. Data were tested for normality by evaluating the Shapiro-Wilk statistic using PROC UNIVARIATE of SAS. The GROUP option in the REPEATED statement was used to separate variances and adjusted for unequal variances if needed. Data were 1/X or log_10_ transformed when needed. Differences between least square means were determined with the PDIFF test of SAS. Significance was declared at *p* < 0.05 and tendency at *p* < 0.10 unless otherwise indicated.

## 3. Results and Discussion

### 3.1. Rectal Temperature and Respiratory Rate

Heat-stressed goats experienced increased (*p* < 0.001) average rectal temperatures (+0.76 °C) and respiratory rates (+76 breaths/min) compared to TN goats (Table 2). Furthermore, values of rectal temperature and respiratory rate increased (*p* < 0.05) in HS goats from 39.27 °C and 81 breaths/min at 08:00 h to 39.79 °C and 111 breaths/min at 12:00 h. Additional increase (*p* < 0.05) was observed at 17:00 h (40.07 °C and 134 breaths/min) in accordance with the increment in the ambient temperature from 30 °C during night to 37 °C during the day. For TN goats, average rectal temperature and respiratory rate values increased (*p* < 0.05) from 38.74 °C and 28 breaths/min at 08:00 h to 39.00 °C and 33 breaths/min at 12:00 h. No additional increase was observed at 17:00 h for TN goats (39.10 °C and 35 breaths/min). These results agree with the findings of Hamzaoui et al. [1], Mehaba et al. [6], and Contreras-Jodar et al. [9], where goats exposed to HS experienced high rectal temperatures and respiratory rates. The increment in respiratory rate under HS conditions was for dissipating the heat load by pulmonary water evaporation as indicated by the greater consumption of water by HS goats (see hereafter).

No effect of PG supplementation on rectal temperature or respiration rate was detected under TN or HS conditions (Table 2). Nielsen and Ingvartsen [11] indicated that farmers and veterinarians in Denmark have observed that some cows had rapid shallow breathing, ataxia, salivation, somnolence, and depression when adding PG to the ration. Rapid shallow breathing, ataxia, and central nervous system depression have also been described as symptoms of toxicity when PG was provided at high doses [22]. The dose used in the current study was not toxic as respiratory rate was not affected by PG supplementation.

### 3.2. Body Weight Change, Feed Intake, and Energy Balance

The BW change, DM intake, water consumption, and calculated energy balance data of TN and HS goats with or without PG supplementation are shown in Table 2. The decrease (*p* < 0.01) in DM intake by the effect of HS was 34% on average, which is similar to DM intake losses in heat-stressed goats at mid lactation (–26%; [6]), but greater than losses observed during late lactation (–19%; [1]). Heat-stressed animals decrease their DM intake to reduce the production of metabolic heat given the fact that heat increment of feeding is an important source of heat production in ruminants [23]. Further, the gut fill by water in HS goats might also be related to the reduced feed intake. Our HS goats almost doubled (+185%) their water consumption, and presumably this was due to the increment in heat dissipation by evaporation (panting and sweating).

In accordance with the decreased feed intake, HS goats experienced negative energy balance (–0.34 Mcal/d on average), whereas the energy balance of TN was positive (1.06 Mcal/d on average). This negative energy balance related to HS was also observed in heat-stressed dairy cows [3]. In accordance with the feed intake and energy balance data, HS goats lost BW (–121 g/d on average), whereas TN goats gained BW (+165 g/d on average) during the experimental period. It should be kept in mind that changes in BW of our TN and HS goats included the inevitable variations in the digestive tract content.

Propylene glycol supplementation tended (*p* < 0.10) to decrease average DM intake values (–9%) with no effect on water consumption. The actual amounts of PG consumed by TN-PG and HS-PG goats were 129 ± 5 and 80 ± 3 g/d, respectively, representing 5.9 and 5.8% of the diet on DM basis. In previous experiments, dairy goats [13] and ewes [24] were supplemented daily with 155 and 80 to 160 g PG, respectively, which is comparable to the levels of PG used in the current experiment. However, no feed intake data were reported in these goats and sheep studies [13,24]. In cattle, Miyoshi et al. [20] observed decreased feed intake after 1 to 2 days of top-dressing 518 g/d of PG (3.4% of DM intake). Further, Dhiman et al. [25] reported a significant reduction in DM intake (–10%) in mid-lactating cows fed a forage-based diet (78% alfalfa silage) supplemented with 5% PG mixed with the ration. Emery et al. [26] indicated that feed intake of nonlactating cows is not decreased until PG intake exceeded 5% of dietary DM. In the current study, we chose this relatively high dose of PG (i.e., 5.8%) to guarantee significant increment in blood glucose (especially that PG was not drenched but given mixed with diet ingredients) as the objective was to increase blood glucose levels and spare body protein by providing an alternative source of carbon for gluconeogenesis.

The reduction in DM intake could be explained by the unpalatability of the PG [11]. In the current study, PG was mixed with the ration, resulting in minimal effects on palatability. Alternatively, the impact of PG on feed intake could be related to the effect of greater blood glucose levels on satiety (more blood glucose in PG goats was observed as indicated hereafter). It has been reported that feed intake of lactating cows is decreased when glucose was infused [27]. Despite the reduced DM intake (and consequently NE intake) in the PG-supplemented goats, their energy balance was improved compared to CO goats due to the fact that PG contains high NE_L_ (4.7 Mcal/kg) and milk energy reduced in PG goats (reduced milk fat content by PG as shown later).

Some degree of confusion between the effects of PG and DM intake could exist because PG tended (*p* < 0.10) to reduce the DM intake in both TN and HS goats (–9% on average). However, the reduced energy intake caused PG (–0.21 and –0.30 Mcal NE_L_/d in TN and HS goats, respectively) was fully made up for by the supplemented NE_L_ from PG. The dietary addition of PG provided TN and HS goats with +0.61 and +0.38 Mcal/d NE_L_, respectively. Therefore, PG supplementation had negligible effects on energy intake. In fact, energy balance as well as BW were improved (*p* < 0.05) in both TN and HS goats supplemented with PG (Table 2). Another concern is the reduced intake of nutrients other than energy (i.e., protein, vitamins, minerals, etc.) when PG was supplemented. However, the current diet was formulated with safety margins for protein level (125% of PDI requirements) in dairy goats [18]. Additionally, goats had free access to mineral and vitamin blocks throughout the experiment.

### 3.3. Milk Yield and Milk Composition

Milk yield, fat-corrected milk, and milk composition (fat, protein, and lactose contents) data of TN and HS dairy goats with or without PG supplementation are shown in Table 2. The HS had no effect on milk yield, which agrees with what was observed in dairy goats by Hamzaoui et al. [1]. The nonsignificant loss in milk yield observed in the current study (–6%) is in the range (3 to 13%) reported by Salama et al. [2] for HS-dairy goats. However, milk fat content (and consequently fat-corrected milk), protein content, lactose content, and yields of milk fat and protein decreased (*p* = 0.009 to 0.074) by HS. Losses in milk components due to HS have been also observed in dairy cows [3] and ewes [28,29]. In addition to the decreased feed intake, HS has direct negative effects on the synthetic capacity of the mammary gland as evidenced by the reduced abundance of genes related to milk fat and protein synthesis in heat-stressed mammary cells [4,5].

The PG supplementation did not affect milk yield but decreased (*p* < 0.01) milk fat content, resulting in lower (*p* < 0.01) fat-corrected milk. No effect of PG on milk protein, lactose or somatic cell count was detected. Our hypothesis was that supplementation with PG would increase blood glucose, reduce the use of glucogenic AA for gluconeogenesis, and consequently spare AA for milk protein synthesis. The PG increased blood glucose (see thereafter) and decreased milk fat (sparing more energy), but neither milk yield nor milk protein were improved by PG. This saved energy was clearly deposited in the body and was not used for milk production as BW gain increased (*p* < 0.05) in TN and HS goats when PG was supplemented (Table 2).

The no change in milk yield observed in our study agrees with other studies, where milk yield in cows supplemented with PG in early lactation was not affected [30]. Nevertheless, cows supplemented with 300 g PG in the concentrate for the first 21 days of lactation tend to have a higher milk yield compared with control cows [31]. The milk fat depression (–23% on average) observed in our PG-supplemented goats agrees with what has been reported in dairy cows [32], although this negative effect was not detected by others [20]. The reduced milk fat by PG could be due to, first, the decrease in plasma NEFA (discussed hereafter) since lowered NEFA concentrations lead to decreased NEFA-uptake by the mammary gland [32]; and second, PG supplementation lowers the molar proportion of acetate in the rumen [33], which may reduce the amount of acetate available for de novo fatty acids synthesis in the mammary gland. Other effects on rumen fermentation and the production of fatty acids intermediates known to inhibit milk fat synthesis could not be excluded.

### 3.4. Blood Metabolites

Blood acid-base balance indicators as well as basal levels of blood insulin, glucose, urea, creatinine, NEFA, BHB, and lactate of TN and HS goats supplemented or not with PG are shown in Table 3. Values of total blood CO_2_, CO_2_ partial pressure, and HCO_3_ were lower (*p* < 0.001) in HS compared to TN goats, which agrees with previous studies [1,6]. The greater respiratory rate observed in HS goats (Table 2) contributed to washing off of CO_2_, and consequently a lower concentration of carbonic acid in blood. As a compensation mechanism to keep blood pH constant, HCO_3_ was transferred from the blood to urine by the kidney [1,34]. This compensation mechanism was not affected by PG supplementation (no significant temperature by PG interaction was detected for blood values of pH or HCO_3_). Blood Cl concentrations increased, whereas Na and HCO_3_ levels decreased in HS compared to TN goats. Calamari et al. [35] reported an inverse relationship between blood HCO_3_ and Cl in dairy cows under TN and HS conditions, which is in accordance with our findings. Blood Na decreased by HS, which disagrees with the findings of Hamzaoui et al. [1] and Mehaba et al. [6] who observed no difference in blood Na between TN and HS goats.

The decrease (*p* < 0.01) in blood urea concentration by HS (Table 3) could be explained by less DM intake, which resulted in less N absorption by the intestine. Despite the reduced feed intake, HS goats kept similar glucose level to TN goats and did not mobilize body fat reserves (no change in NEFA or BHB values). Similarly, Hamzaoui et al. [1] and Mehaba et al. [6] reported that blood insulin, glucose, and NEFA do not vary between TN and HS goats. In dairy cows, Baumgard and Rhoads [3] reported that blood NEFA did not vary due to HS (similar to goats), but blood insulin levels were significantly increased (such an increase was not detected in goats). Furthermore, Baumgard and Rhoads [3] indicated in their review on the effects of HS on metabolism and energetics that blood lactate levels are consistently elevated in many HS models, including cattle. The origin of this lactate is unknown but may include the gastrointestinal tract and muscle. Nonetheless, no effect of HS on blood lactate was observed in our goats.

Propylene glycol supplementation had no effect of blood electrolytes or any other indicators of acid-base balance (Table 3). The tendency of greater (*p* < 0.10) blood pH by PG supplementation is of minor physiological importance as blood pH values for all treatment groups were within the normal blood pH values for most mammals (7.35 to 7.45; [36]). Additionally, blood hematocrit and hemoglobin values were not affected by PG. Similarly, periparturient dairy cows given 300 mL of PG (as a drench once daily for 3 days) have similar blood levels of Na, Cl, Ca, K, hematocrit, and hemoglobin compared with control cows [37]. Toxic effects of PG including hemoglobinuria have been reported after i.v. injection of an aqueous solution of PG in sheep [38]. However, our data suggest that PG supplementation at 5.8% of DM intake does not have clinically relevant detrimental effects on blood acid-base balance, hematocrit, or hemoglobin in dairy goats.

The PG supplementation increased (*p* < 0.05) blood glucose levels, and this increment of glucose was accompanied by hyperinsulinemia as blood insulin tended (*p* < 0.10) to increase (Table 3). The PG is a glucogenic precursor that is partially absorbed from the rumen and oxidized in the liver to lactate, which is later converted to glucose by gluconeogenesis [33]. Alternatively, PG is fermented in the rumen to propionate, which increases the molar percentage of propionate with no reduction in total ruminal VFA concentration [11]. Consequently, this propionate is converted into glucose in the liver by gluconeogenesis. Compared to the study of Kristensen and Raun [33], in which PG was orally drenched to cows, PG in the current study was mixed with the ration, which resulted in introducing the PG into the rumen continuously in small quantities throughout the day. This might have given enough time for PG to be fully fermented in the rumen, and no PG escaped to the liver. This might explain why we observed greater blood glucose (presumably from propionate in the rumen) with no change in blood lactate values.

As a result of feeding PG, blood insulin increased in both TN (+35%) and HS (+38%) goats to a similar extent, but glucose increment was greater in TN (+9%) than HS (+4%) goats (*p* of interaction = 0.096; Table 3). It is intriguing that HS goats had less increment in blood glucose although blood insulin increased similarly in both groups. This could be explained by greater sensitivity to insulin under HS conditions. However, by comparing TN and HS animals fed ad libitum (as in the current study), we found that insulin sensitivity is not affected, or even tends to decrease, in heat-stressed goats [2] and ewes [29]. This might indicate greater importance of non-insulin-mediated glucose uptake process. In fact, the whole-body utilization of glucose can also happen by noninsulin-dependent pathways [39]. It is possible that glucose from PG in HS goats was rapidly taken up by insulin- and noninsulin-dependent pathways and directed to alleviate BW losses rather than milk synthesis (HS-PG goats lost less BW than HS-CO goats). In addition to its use in muscles, the extra glucose due to PG supplementation would have been used by immune cells as HS is known to cause inflammation as mentioned above [3,9]. Unfortunately, no inflammation markers were measured in the current experiment.

In addition to its effects on glucose and insulin, PG supplementation decreased (*p* < 0.05) NEFA (–49%) and BHB (–32%) in plasma, indicating significant influence of PG on bioenergetics. Considering the lipogenic effect of insulin, it is reasonable to think that PG-induced insulin secretion is responsible for lower (*p* < 0.05) blood NEFA levels, and hence, a decrease (*p* < 0.01) in BHB concentration in goats fed PG. Finally, creatinine concentration was greater (*p* < 0.05) in goats fed PG. Goats fed hay supplemented with PG also experience greater blood creatinine levels [13].

## 4. Conclusions

Propylene glycol did not affect milk yield or milk protein content, despite the increment in circulating blood glucose and insulin. Furthermore, a strong milk fat depression was detected when propylene glycol was supplemented. Propylene glycol also influenced animal bioenergetics as evidenced by decreased blood levels of non-esterified fatty acids and β-hydroxybutyrate. Nevertheless, dietary propylene glycol alleviated body weight loss typically observed in heat-stressed goats. Future research is needed to test whether lower doses of propylene glycol would avoid reduced feed intake and milk fat depression, while keeping the positive effect on blood glucose and body weight under heat stress conditions.

## Figures and Tables

**Table 1:** Chemical composition and nutritive value of the control ration expressed on dry matter basis.

Item	Total Mixed Ration
Component, %	
Dry matter	89.5
Organic matter	87.6
Crude protein	16.4
Neutral detergent fiber	35.8
Acid detergent fiber	24.6
Nutritive value ^1^	
UFL,^2^ /kg	0.82
NE_L_, Mcal/kg	1.43
PDI,^3^ g/kg	100
PDIA,^4^ g/kg	50.0
Calcium, g/kg	10.6
Phosphorous, g/kg	2.86

^1^ Calculated according to the French National Institute for Agricultural Research (INRA) [18]. ^2^ Forage unit for lactation (1 UFL = 1.76 Mcal of NE_L_). ^3^ Protein digestible in the intestine from dietary and microbial origin. ^4^ Protein digestible in the intestine from dietary origin.

**Table 2:** Least squares means for physiological variables, feed intake, and milk production of dairy goats under thermoneutral (TN) and heat stress (HS) conditions. In each ambient temperature, goats were fed a control diet (CO) or supplemented with propylene glycol (PG). ^1.^

Variable	TN		HS		SEM	Effect ^2^ (*p* =)		
	CO (*n* = 8)	PG (*n* = 8)	CO (*n* = 8)	PG (*n* = 8)		T	S	T × S
Rectal temperature, °C	38.93	38.97	39.76	39.66	0.05	0.001	0.315	0.209
Respiratory rate, breaths/min	31	32	109	107	2	0.001	0.432	0.211
Body weight change, kg	2.14	4.13	–2.93	–1.66	0.79	0.001	0.049	0.649
Dry matter intake, kg/d	2.34	2.19	1.59	1.38	0.07	0.001	0.060	0.776
Energy balance, Mcal/d	0.79	1.32	–0.50	–0.17	0.11	0.001	0.001	0.376
Water consumption, L/d	5.99	5.84	11.16	10.74	1.07	0.001	0.797	0.900
Milk yield, kg/d	1.86	1.80	1.79	1.66	0.18	0.210	0.258	0.614
Fat-corrected milk, L/d ^3^	2.12	1.78	1.85	1.48	0.16	0.002	0.001	0.856
Milk composition, %								
Fat	4.43	3.46	3.78	2.89	0.15	0.009	0.002	0.856
Protein	3.55	3.54	3.14	3.15	0.15	0.074	0.994	0.963
Lactose	4.47	4.46	4.31	4.29	0.06	0.064	0.886	0.980
Fat: Protein ratio	1.26	1.00	1.21	0.93	0.06	0.365	0.001	0.876
Fat yield, g/d	79.9	62.8	67.2	48.2	5.7	0.025	0.004	0.870
Protein yield, g/d	63.7	61.8	55.8	52.0	4.4	0.054	0.521	0.832
Lactose yield, g/d	81.8	82.5	78.0	72.9	8.6	0.443	0.794	0.738
Somatic cell count, Log_10_	5.66	5.67	5.97	5.82	0.18	0.231	0.708	0.652

^1^ Each goat received one treatment (TN-CO, TN-PG, HS-CO, HS-PG) in one of the 4 experimental period (14 days for adaptation and 5 days for measurements). The data shown for each treatment are the average of the experimental days of the 4 periods. ^2^ Effects of ambient temperature (T), PG supplementation (S), and their interaction (T × S). ^3^ Fat corrected milk at 3.5%; fat-corrected milk = kg of milk yield × [0.432 + 0.162 × (fat%)].

**Table 3:** Least squares means for blood metabolites in dairy goats under thermoneutral (TN) and heat stress (HS) conditions. In each ambient temperature, goats were fed a control diet (CO) or supplemented with propylene glycol (PG). ^1^

Variable	TN		HS		SEM	Effect ^2^ (*p* =)		
	CO (*n* = 8)	PG (*n* = 8)	CO (*n* = 8)	PG (*n* = 8)		T	S	T × S
pH	7.42	7.44	7.43	7.45	0.01	0.206	0.093	0.965
Na, mmol/L	150	151	148	149	1	0.019	0.223	0.786
K, mmol/L	3.77	3.73	3.96	3.73	0.12	0.463	0.282	0.466
Cl, mmol/L	111	112	114	113	1	0.033	0.999	0.496
Ionized Ca, mmol/L	0.91	0.92	0.94	0.88	0.05	0.919	0.703	0.581
Total CO_2_, mmol/L	25.4	26.4	21.0	20.9	0.8	0.001	0.591	0.497
CO_2_ partial pressure, mmHg	39.6	39.4	28.4	29.5	1.3	0.001	0.715	0.578
O_2_ partial pressure, mmHg	24.1	25.3	26.2	27.1	1.4	0.214	0.436	0.882
HCO_3_, mmol/L	25.5	26.5	19.0	20.4	0.8	0.001	0.243	0.758
Anion gap	19.8	18.9	19.4	21.4	1.2	0.209	0.467	0.078
Hematocrit, %PCV	18.4	19.0	18.1	17.6	1.2	0.484	0.991	0.640
Hemoglobin, g/dL	6.27	6.43	6.17	5.99	0.41	0.513	0.972	0.678
Insulin, µg/L	1.14	1.54	1.03	1.43	0.16	0.674	0.098	0.985
Glucose, mg/dL	56.2	61.5	55.8	58.2	1.5	0.219	0.033	0.096
Blood urea N, mg/dL	25.7	23.9	18.4	18.1	2.8	0.007	0.628	0.722
Creatinine, mg/dL	0.49	0.53	0.49	0.57	0.04	0.490	0.049	0.281
Non-esterified fatty acids, mmol/L	0.10	0.07	0.08	0.04	0.01	0.351	0.032	0.898
β-hydroxybutyrate, mmol/L	0.65	0.48	0.77	0.48	0.05	0.368	0.002	0.397
Lactate, mmol/L	0.51	0.52	0.46	0.51	0.03	0.490	0.446	0.602

^1^ Each goat received one treatment (TN-CO, TN-PG, HS-CO, HS-PG) in one of the 4 experimental periods (14 days for adaptation and 5 days for measurements). The data shown for each treatment are the average of the experimental days of the 4 periods. ^2^ Effects of ambient temperature (T), PG supplementation (S), and their interaction (T × S).
